# Rivaroxaban versus aspirin in prevention of venous thromboembolism following total joint arthroplasty or hip fracture surgery: a meta-analysis

**DOI:** 10.1186/s13018-021-02274-z

**Published:** 2021-02-13

**Authors:** Bangsheng Hu, Lianxiang Jiang, Haixia Tang, Meizhu Hu, Jun Yu, Zeping Dai

**Affiliations:** 1 Department of Anesthesiology, The First Affiliated Hospital of Wannan Medical College, No.2, West Road of Zheshan, Wuhu, 241000 Anhui China; 2 Department of Cardiology, The First Affiliated Hospital of Wannan Medical College, Wuhu, Anhui China

**Keywords:** Rivaroxaban, Aspirin, Thromboembolism, Arthroplasty, Meta-analysis

## Abstract

**Objective:**

To evaluates the efficacy and safety of rivaroxaban versus aspirin in prevention of venous thromboembolism (VTE) following total hip (THA) or knee arthroplasty (TKA) or hip fracture surgery.

**Methods:**

Major databases were systematically searched for all relevant studies published in English up to October 2020. The meta-analysis was conducted using RevMan 5.3 software.

**Results:**

In total, 7 studies were retrieved which contained 5133 patients. Among these patients, 2605 patients (50.8%) received rivaroxaban, whereas 2528 patients (49.2%) received aspirin. There were no statistical difference between aspirin and rivaroxaban for reducing VTE (RR = 0.75, 95% CI 0.50–1.11, *I*^2^ = 36%, *p* = 0.15), major bleeding (RR = 0.94, 95% CI 0.45–2.37, *I*^2^ = 21%, *p* = 0.95), and all-cause mortality (RR = 0.88, 95% CI 0.12–6.44, *I*^2^ = 0%, *p* = 0.90) between the two groups. Compared with aspirin, rivaroxaban significantly increased nonmajor bleeding (RR = 1.29, 95% CI 1.05–1.58, *I*^2^ = 0%, *p* = 0.02).

**Conclusion:**

There was no significant difference between aspirin and rivaroxaban in prevention of venous thromboembolism following total joint arthroplasty or hip fracture surgery. Aspirin may be an effective, safe, convenient, and cheap alternative for prevention of VTE. Further large randomized studies are required to confirm these findings.

**Supplementary Information:**

The online version contains supplementary material available at 10.1186/s13018-021-02274-z.

## Introduction

Venous thromboembolism (VTE) is a serious perioperative complication and one of the main causes of unintended death during perioperative period [1–4]. VTE, including deep vein thrombosi (DVT) and pulmonary embolism (PE), is one of the well-recognized complications after total joint arthroplasty. Virchow, a German physiologist of the last century, believed that a clot is a change in the nature of the blood in a blood vessel under certain conditions [5]. In various pathological conditions, abnormal activation of the coagulation system in the blood can lead to the formation of emboli. Currently, there are three recognized factors of thrombosis, that is, the damage of vessel wall, abnormal blood flow, and abnormal coagulation system. Epidemiological studies have shown that more than half of hospitalized patients worldwide are at risk for venous thromboembolism [6]. Currently, the main clinical anticoagulants used include direct oral anticoagulants like rivaroxaban, a factor Xa inhibitor, and enoxaparin, a low molecular weight heparin (LMWH). However, it is a controversial topic since aspirin has emerged for a few years as a potential cost-effectiveness alternative for the prevention of thrombosis during arthroplasty.

Rivaroxaban is an oral highly selective factor Xa blocker that competitively inhibits both free and bound factor Xa and prothrombin activity, thus effectively and safely preventing deep vein thrombosis [7]. In recent years, it has been clinically used in many countries due to its good anticoagulant effect. Aspirin is a cheap, universal, and widely available antiplatelet drug. The efficacy of aspirin in prevention of cardiovascular and cerebrovascular ischemic diseases has been proved, but whether aspirin should be used as a routine drug for prevention of VTE after surgery is still controversial [8, 9].

Thus, studies of clinical trials conducted to compare the effects of rivaroxaban versus aspirin in preventing VTE. We conducted a meta-analysis to evaluate the efficacy and safety of rivaroxaban versus aspirin in prevention of VTE after total hip (THA) or total knee arthroplasty (TKA) or hip fracture surgery.

We present the following article in accordance with the PRISMA Reporting Checklist. And the PRISMA Reporting Checklist is provided in the Supplementary files.

## Materials and methods

### Methodology

We searched PubMed, Embase, the Cochrane Library, and EBSCO. Reviews, meetings, case reports, and related references in eligible studies were also included. Limits were set to literatures published in English up to October 2020. RevMan 5.3 software was used to carry out the meta-analysis. The search terms included “rivaroxaban”, “aspirin”, “venous thromboembolism”, “bleeding”, “perioperative period”, “arthroplasty”, “replacement”, and “anticoagulants”.

### Selection criteria

The following standards were required for the included studies: (1) patients receiving rivaroxaban and aspirin after total joint arthroplasty or hip fracture surgery, (2) primary endpoint outcome was VTE and bleeding events, and (3) studies only published in English. The exclusion criteria consisted of the following: (1) excluding literature reviews, case reports, animal experiments, laboratory studies, and repeated publications and (2) excluding information to provide incomplete literature.

### Data extraction and management

Study search, selection, abstraction, and quality assessment were all performed by two independent reviewers and all disparate opinions were resolved through discussion.

### Assessment of the quality of the studies

The methodological quality of the included studies was estimated independently by two authors based on The Cochrane Risk of Bias criteria. Each quality item was graded as low risk, high risk, or no clear risk. The seven items used to assess bias in each trial included randomization sequence generation, allocation concealment, blinding of participants and personnel, blinding of outcome assessment, incomplete outcome data, selective reporting, and other biases.

### Outcomes

The primary effectiveness outcome was adjudicated symptomatic venous thromboembolism, which was defined as DVT involving the popliteal vein or more proximal leg veins (including the femoral, common femoral, and iliac veins and inferior vena cava) or PE. The primary safety outcome was bleeding, including major or clinically relevant nonmajor bleeding. Secondary outcome measures were all-cause mortality.

### Statistical analysis

RevMan 5.3 software was used to analyze the extracted data. Relative risk (RR) was used as effect size for counting data, and each effect size and its 95% confidence interval (CI) were used to represent the results. The included studies were first tested for clinical heterogeneity. If there was no heterogeneity between the studies (*I*^2^ < 50%), the fixed effect model was selected for meta-analysis. On the contrary, there was heterogeneity among the studies (*I*^2^ > 50%); the reasons for the heterogeneity were analyzed, and factors of heterogeneity were analyzed using subgroups. If there was statistical heterogeneity between the two groups, but no clinical heterogeneity or statistical difference, the random effect model was used for analysis. Descriptive analysis was used when the heterogeneity of the two groups was too large or data sources could not be found. Funnel plot was used to indicate the publication bias.

## Results

### Study selection process

**Fig. 1:**
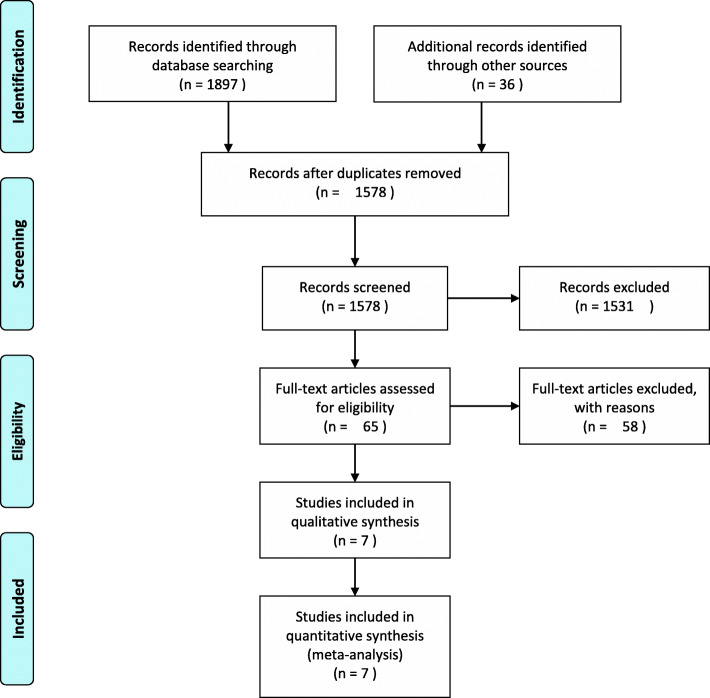
PRISMA flowchart of the selection process

In this study, 1933 related literatures were initially retrieved. After reviewing the titles and abstracts, 1513 obviously unrelated literatures were excluded and 65 literatures were enrolled. Through full text reading, a total of 65 republished literatures and literatures with no research results were again excluded, and 7 studies [4, 10–15] were finally included, with a total of 5133 patients. Among the 7 studies, there were 2605 patients in the rivaroxaban group and 2528 patients in the aspirin group. PRISMA flowchart of the selection process is shown in Fig. 1.

### Characteristics of the eligible studies

In this study, the year of publication, country, number of cases, postoperative drug use, and other basic information in the included literature were extracted, and the general characteristics of the included study were shown in Table 1.

**Table 1:** Characteristics of studies included in the meta-analysis

First author	Year	Country	Sample number (rivaroxaban/aspirin)	Average age (years) (rivaroxaban/aspirin)	Type of surgery	Intervention (rivaroxaban/aspirin)	Follow-up	Endpoints
Jiang [10]	2014	China	60/60	63.8 ± 6.7/65.1 ± 7.5	TKA	10 mg once daily, 14 days/100 mg once daily, 14 days	42 days	VTE
Zou [11]	2014	China	102/110	63.50/62.70	TKA	10 mg once daily, 14 days/100 mg once daily, 14 days	28 days	VTE, nonmajor bleeding
Colleoni [12]	2018	Brazil	18/14	67.11 ± 7.65/71.21 ± 6.35	TKA	10 mg once daily, 14 days/300 mg once daily, 14 days	90 days	VTE, all-cause mortality
Anderson (a) [4]	2018	Canada	902/902	60.90 ± 11.00/61.30 ± 11.10	THA	10 mg once daily, 30 days/81 mg once daily, 30 days	90 days	VTE, major bleeding, nonmajor bleeding, all-cause mortality
Anderson (b) [4]	2018	Canada	815/805	64.70 ± 8.40/64.60 ± 8.70	TKA	10 mg once daily, 9 days/81 mg once daily, 9 days	90 days	VTE, major bleeding, nonmajor bleeding, all-cause mortality
Lindquist [13]	2018	USA	440/360	65.4/65.8	THA or TKA	10 mg once daily, THA: 35 days, TKA: 12 days/325 mg twice daily, THA: 35 days, TKA: 12 days	30 days	major bleeding, nonmajor bleeding
Yuenyongviwat [14]	2019	Thailand	76/79	71.41 ± 6.17/70.08 ± 5.22	TKA	10 mg once daily, 14 days/100 mg once daily, 14 days	42 days	VTE, major bleeding, nonmajor bleeding
Huang [15]	2019	China	192/198	67.8 ± 16.9/69.4 ± 17.4	HFS	10 mg once daily, 14 days/100 mg once daily, 16 days	90 days	VTE, major bleeding, nonmajor bleeding

*TKA* Total knee arthroplasty, *THA* Total hip arthroplasty, *HFS* Hip fracture surgery, *VTE* Venous thromboembolism, *Anderson (a)* the type of surgery in this study was THA, *Anderson (b)* the type of surgery was TKA

### Quality of the eligible studies

A total of 7 literatures were included in this meta-analysis, among which 5 trials [4, 10–12, 15] were randomized controlled trials (RCTs) and 2 trials [13, 14] were retrospective studies. In all the five RCT studies, reasonable randomization methods and hidden allocation methods were given, and in one of them, double-blind methods were used, with a low risk of bias. The quality evaluation table of literature is shown in Table 2.

**Table 2:** Risk of bias assessment for included studies according to the Cochrane Collaboration’s tool

First author	Random allocation	Hidden distribution	Blind method	Incomplete outcome data	Selective reporting of results	Other bias
Jiang [10]	Randomized	No clear	No clear	Low	Low	Low
Zou [11]	Randomized	No clear	No clear	Low	Low	Low
Colleoni [12]	Randomized	No clear	No clear	Low	Low	Low
Anderson [4]	Randomized	No clear	Double-blind	Low	Low	Low
Lindquist [13]	No clear	No clear	No clear	Low	Low	Low
Yuenyongviwat [14]	No clear	No clear	No clear	Low	Low	Low
Huang [15]	Randomized	No clear	No clear	Low	Low	Low

## Results of the meta-analysis for outcomes

### The incidence of VTE

**Fig. 2:**
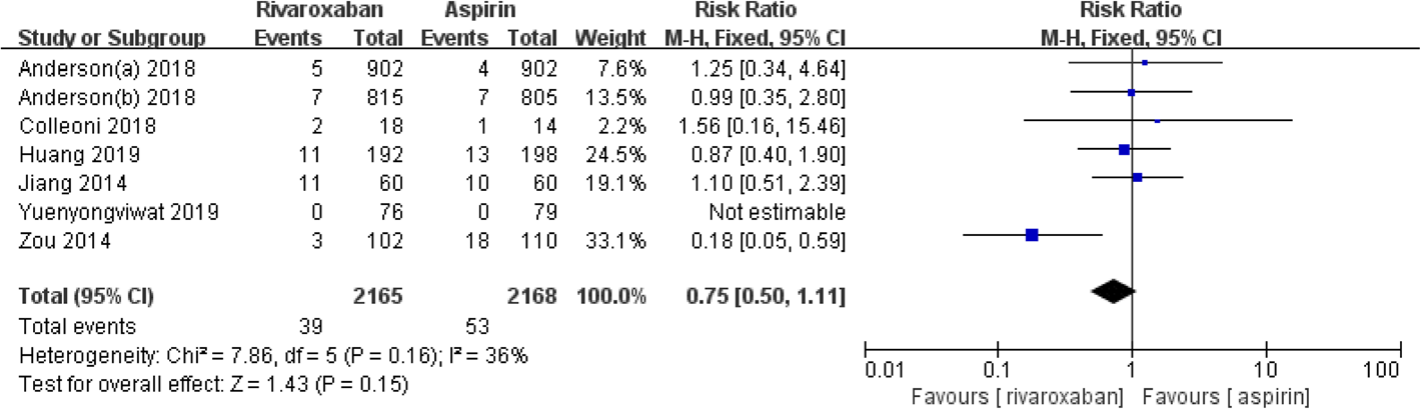
Forest plot of venous thromboembolism (VTE)

Six studies including 4333 patients compared the incidence of perioperative VTE events between the rivaroxaban and aspirin groups [4, 10–12, 14, 15]. The meta-analysis showed that there was no statistically significant difference in the incidence of postoperative VTE between the two groups (RR = 0.75, 95% CI 0.50–1.11, *I*^2^ = 36%, *p* = 0.15), as shown in Fig. 2.

### Major bleeding

**Fig. 3:**
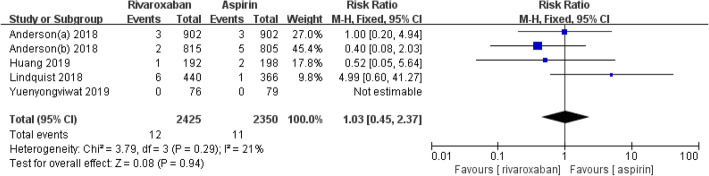
Forest plot of major bleeding

Four studies with 4775 participants reported data for major bleeding [4, 13–15]. In total, 12 out of 2425 patients in the rivaroxaban group experienced major bleeding, while 11 out of 2350 patients in the aspirin group experienced major bleeding. The results showed that there were no significant differences in major bleeding between the rivaroxaban and aspirin groups (RR = 0.94, 95% CI 0.45–2.37, *I*^2^ = 21%, *p* = 0.94), as shown in Fig. 3.

### Nonmajor bleeding

**Fig. 4:**
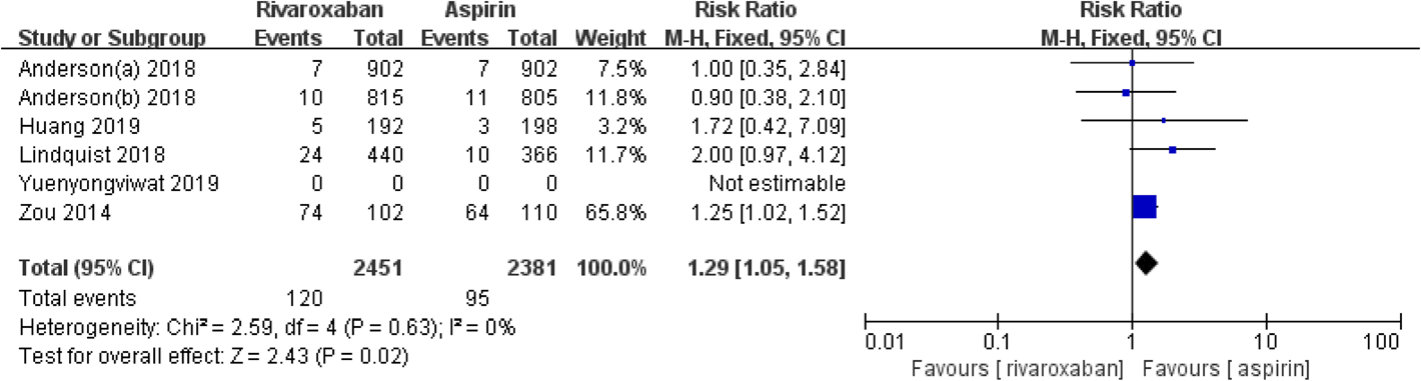
Forest plot of nonmajor bleeding

Five studies with 4832 patients reported data for the incidence of nonmajor bleeding [4, 11, 13–15]. The analysis showed that rivaroxaban increased the incidence of nonmajor bleeding compared with aspirin (RR = 1.29, 95% CI 1.05–1.58, *I*^2^ = 0%, *p* = 0.02), as shown in Fig. 4.

### All-cause mortality

**Fig. 5:**
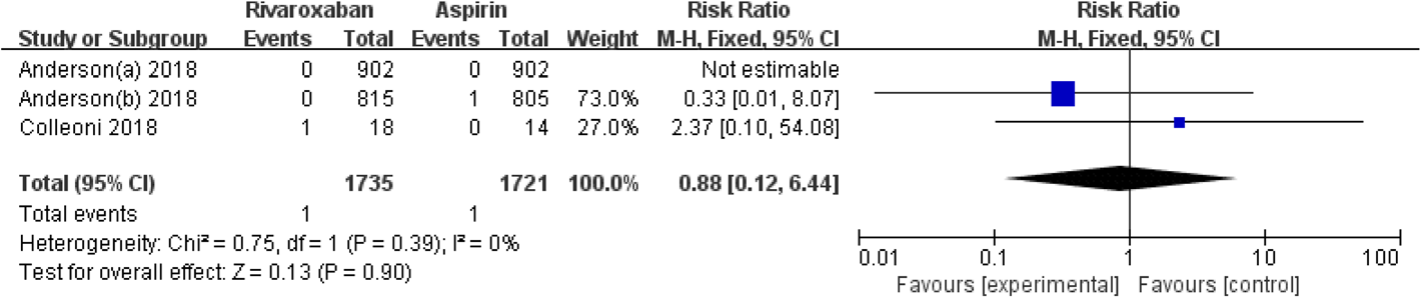
Forest plot of all-cause mortality

Two studies, with 3456 enrolled participants, reported the all-cause mortality in the two groups [4, 12]. The results showed that there was no significant difference in the all-cause mortality between the two groups (RR = 0.88, 95% CI 0.12–6.44, *I*^2^ = 0, *p* = 0.90), as shown in Fig. 5.

## Discussion

We found that 7 studies evaluated the efficacy and safety of rivaroxaban versus aspirin in prevention of VTE following total joint arthroplasty or hip fracture surgery. The results demonstrated that there were no statistical difference between rivaroxaban and aspirin in prevention of VTE and the reduction of major bleeding and all-cause mortality. However, rivaroxaban had some negative side effects to patients such as nonmajor bleeding.

VTE is the most common cause of perioperative hospitalization death, and its complications consume a large amount of medical resources [6, 16]. Anticoagulant drugs have been shown to reduce postoperative mortality and complications associated with VTE [17]. We reasoned that aspirin, because of its efficacy, low cost, and well-established side-effect profile, was potentially a good choice for thromboprophylaxis after total hip or total knee arthroplasty or hip fracture surgery [4, 18]. Rivaroxaban is the world’s first oral inhibitor of factor Xa. It is used to prevent and treat venous thromboembolism and prevent stroke or systemic embolism in atrial fibrillation [19]. It has advantages of convenient administration, rapid action, and low risk of drug interaction. In recent years, there have been more and more studies on rivaroxaban in perioperative anticoagulation, especially in prevention of VTE in patients with lower limb fractures. The use of oral anticoagulants in the perioperative period, represented by rivaroxaban, seems to be in doubt. To date, clinical trials have suggested that aspirin may be effective for prevention of venous thromboembolism postoperatively, but comparisons with direct oral anticoagulants are lacking. We pooled the patient cohort from eight studies to increase the power of these findings. In addition, we hope to provide more objective evidence for prevention of VTE.

In fact, the recent study published by Weitz et al. [19], which compares the efficacy and safety of rivaroxaban (10 mg/day and 20 mg/day) with aspirin in secondary prevention of VTE demonstrates the absolute superiority of the anticoagulant in reducing the incidence of thromboembolic events, without increasing bleeding. In addition, a recent meta-analysis has shown a benefit for prevention with rivaroxaban [20]. However, the inclusion criteria are not all surgical patients. Our study included patients receiving rivaroxaban and aspirin after surgery. It is well known that perioperative period is a crucial period with a high risk of VTE event but also with high risk for bleeding on the operative zone. As a matter of fact, the American Association of Orthopaedic Surgery recommends aspirin as a chemoprophylactic drug for VTE in 2012 [21]. In addition, other recent studies have shown that aspirin did not differ statistically significantly from other anticoagulants used for VTE prophylaxis after THA and TKA [22–24]. These studies support our conclusion.

In this meta-analysis, the incidence of postoperative VTE, the risk of bleeding, and all-cause mortality were compared. Compared with rivaroxaban, aspirin had no statistical difference in prevention of perioperative venous thromboembolism. Thus, our findings suggest that aspirin may be an appropriate option, which is not only cost-effective, but also has a similar ability to prevent VTE following total joint arthroplasty or hip fracture surgery.

This meta-analysis included five randomized controlled studies and two cohort studies involving a large sample of 5133 patients. These literatures were published in high-level journals, five literatures of which were published in recent 3 years, and the results were reliable. There have been numerous studies on prevention of VTE with anticoagulants, including comparisons of enoxaparin with rivaroxaban, rivaroxaban with placebo, and aspirin with placebo, with very few studies comparing rivaroxaban with aspirin. This study collected recent studies on rivaroxaban and aspirin, and comprehensively analyzed the efficacy and safety of these two drugs in prevention of perioperative VTE. With more and more people supporting cheaper aspirin, it makes sense to include all relevant studies in recent years in a meta-analysis.

This meta-analysis also has some limitations. (1) Although this study adopts a comprehensive retrieval strategy, it is still possible to miss some gray literature. (2) The sample size varied greatly among the included studies, which ranged from 32 to 3424. Compared with larger trials, studies with small sample size were more likely to produce an overestimated treatment effect. (3) Only one study has described the use of blind methods, which may have a mixed bias. (4) When analyzing the bleeding events caused by the two drugs, we used nonmajor bleeding events instead of events other than major bleeding, and such differences may affect our final results. (5) There are also many influencing factors for the mortality of patients. Some confounding factors cannot be completely avoided in this study. (6) This study did not specifically analyze the basic diseases and medical history of the study patients. Aspirin has been shown to increase the risk of stomach bleeding. Therefore, aspirin should be used with caution in a patient with a history of stomach ulcers.

## Conclusion

The results of this meta-study found aspirin no significant difference in the efficacy and safety of aspirin in prevention of VTE when compared with rivaroxaban. Aspirin may be an effective, safe, convenient, and cheap alternative for prevention of VTE following total joint arthroplasty or hip fracture surgery. Our study needs to be validated with more high-quality, large-sample RCTs.

## Supplementary Information

**Additional file 1:.** PRISMA 2009 Checklist.

## Data Availability

The datasets used and analyzed during the current study are available from the corresponding author on reasonable request.

## Abbreviations

VTE: Venous thromboembolis
TKA: Total knee arthroplasty
THA: Total hip arthroplasty
CI: Confidence interval
RR: Risk ratio
DVT: Deep vein thrombosis
PE: Pulmonary embolism
LMWH: Low molecular weight heparin
HFS: Hip fracture surgery
RCTs: Randomized controlled trials
